# Blueberry and Mulberry Juice Prevent Obesity Development in C57BL/6 Mice

**DOI:** 10.1371/journal.pone.0077585

**Published:** 2013-10-15

**Authors:** Tao Wu, Qiong Tang, Zichun Gao, Zhuoping Yu, Haizhao Song, Xiaodong Zheng, Wei Chen

**Affiliations:** 1 College of Biosystems Engineering and Food Science, Zhejiang University, Hangzhou, Zhejiang, China; 2 Fuli Institute of Food Science, Zhejiang University, Hangzhou, Zhejiang, China; University of Sassari, Italy

## Abstract

**Objectives:**

To establish whether blueberry (*Vaccinium ashei*) and mulberry (*Morus australis* Poir) juice, anthocyanin rich fruit juice, may help counteract obesity.

**Design:**

**And Methods**: Four-week-old C57BL/6 mice were fed a high-fat diet (HFD) with or without blueberry and mulberry juice for 12 weeks. Body weight, serum and hepatic lipids, liver and adipose tissues morphology, insulin and leptin were assessed.

**Results:**

Mice fed HFD exhibited increased body weight, insulin resistance, serum and hepatic lipids. In comparison, blueberry and mulberry juice inhibited body weight gain, decreased the serum cholesterol, reduced the resistance to insulin, attenuated lipid accumulation and decreased the leptin secretin.

**Conclusion:**

These results indicate that blueberry and mulberry juice may help counteract obesity.

## Introduction

 Obesity confers a myriad of detrimental effect on health and is recognized as a leading global health problem [1]. It is known to contribute to the risk of various chronic diseases such as type II diabetes mellitus, coronary heart disease, hypertension and several types of cancers [2,3]. In addition, it also causes excessive fat accumulation in adipose tissue, leading to hypertrophy of adipocytes and elevated levels of adipocytokine [4]. Currently, there are some therapeutic approaches for treating obesity, such as appetite suppression, self-control and decrease absorption, etc [5]. However, these approaches are often accompanied by severe side-effect and high rates of secondary failure [6]. 

 Anthocyanins are water-soluble pigments widely found in fruits and vegetables and, therefore, often consumed in a normal diet [7]. Furthermore, anthocyanins have been attributed several putative therapeutic roles, including beneficial effects on obesity and related metabolic complications [8,9]. In this respect, anthocyanins from purple corn (*Zea mays* L.) [10], blood orange (*Citrussinensis* L.Osbeck) [11], strawberries (*Fragaria ananassa*) [12,13], blueberries (*Vaccinium angustifolium*) [13,14], blackberries (*Rubus* sp.) [15] and mulberry (*Morus australis* P.) [16] prevent the development of obesity in mice fed a high-fat diet (HFD).

Blueberry and mulberry juices are very popular in recent years as a result of the health benefits associated with the nutritious phytochemicals, especially rich in anthocyanins [13,14,16,17]. Some studies have reported that consumption of 100% fruit juice increase the risk of obesity [18] while several studies have shown that intake of 100% juice inhibit the development of obesity [19]. Therefore, it is very important to clearly identify the effect of mulberry and blueberry juice on the development of obesity. In this study, our purpose was to explore the effect of the administration of blueberry and mulberry juice on the development of obesity in mice fed a high fat diet (HFD).

## Materials and Methods

### Juice preparation

 Fresh blueberry and mulberry were obtained from the agricultural logistics center in Hangzhou. The obtained fruits were immediately stored at 4 °C and few days later extracted using a household juicer, and subsequently centrifuged at 10,000 g for 60 min to pellet down the particulate matter. The obtained juice was filtered and stored in aliquots at -80 °C. The total polyphenols of blueberry and mulberry juice were analyzed according to a Folin-Ciocalteu assay [20]. The anthocyanins of juices were characterized using HPLC/ESI/MS/MS (Agilent 1290 Infinity LC, coupled to a 6400 series triple quadrupole mass spectrometer) and the concentration of each identified anthocyanin was determined by using UltiMate 3000 series HPLC.

### Animals and Animal Care

 All the experimental procedures were conducted in conformity with protocols approved by the Committee on the Ethics of Animal Experiments of Zhejiang University (Permit Number: Zju2012020112) and according to the National Institutes of Health Guide for Care and Use of Laboratory Animals. 

Forty-eight male C57BL/6 mice were purchased from the Branch of National Breeder Center of Rodents (Shanghai, China) at 4 weeks of age and kept in a specific pathogen free facility. They were permanently kept in individual cage under 12h- light/12h-dark cycle and fed ad libitum during the overall experiment. After 7-day adaptation, mice were then split into four groups and fed specific diets for a period of 12 weeks. The groups included: (1) twelve mice fed low-fat diet (provided 3.85 kcal/g with 10% fat, 20% proteins and 70% carbohydrates) and permitted ad libitum consumption of water (LFD + Water); (2) twelve mice fed high-fat diet (provided 4.73 kcal/g with 45% fat, 20% proteins and 35% carbohydrates; Medicience Ltd, China) and permitted ad libitum consumption of water (HFD + Water); (3) twelve mice fed high-fat diet and permitted ad libitum consumption of blueberry juice instead of water (HFD +BBJ); (4) twelve mice fed high-fat diet and permitted ad libitum consumption of mulberry juice instead of water (HFD +MBJ). After 12 weeks, the mice were sacrificed by decapitation. Blood samples, heart, liver, kidney and adipose tissue were collected, weighted and then stored at -80 °C. 

 Body weight and food consumption measurements started the first week of the study (5 weeks of age) and continued weekly for the entire experiment of each mouse. Food consumption was determined for each of the four groups by weighing the total amount of food given at the start of each week and then subtracting by the amount of food remaining at the end of week. The average food consumed per mouse was then obtained by dividing the number of the mice. 

### Serum analysis

 Concentrations of serum glucose, triglycerides, and cholesterol were determined by enzymatic methods using commercially available kits (Elabscience). Serum insulin leptin and adiponectin levels were analyzed by immunoassay using a rat/mouse ELISA kit (R&D system) according to the manufacturer’s protocols. A homeostatic model of insulin resistance (HOMA-IR) was assessed based on insulin and glucose levels obtained according to a previously described method [21].

**Figure 1:**
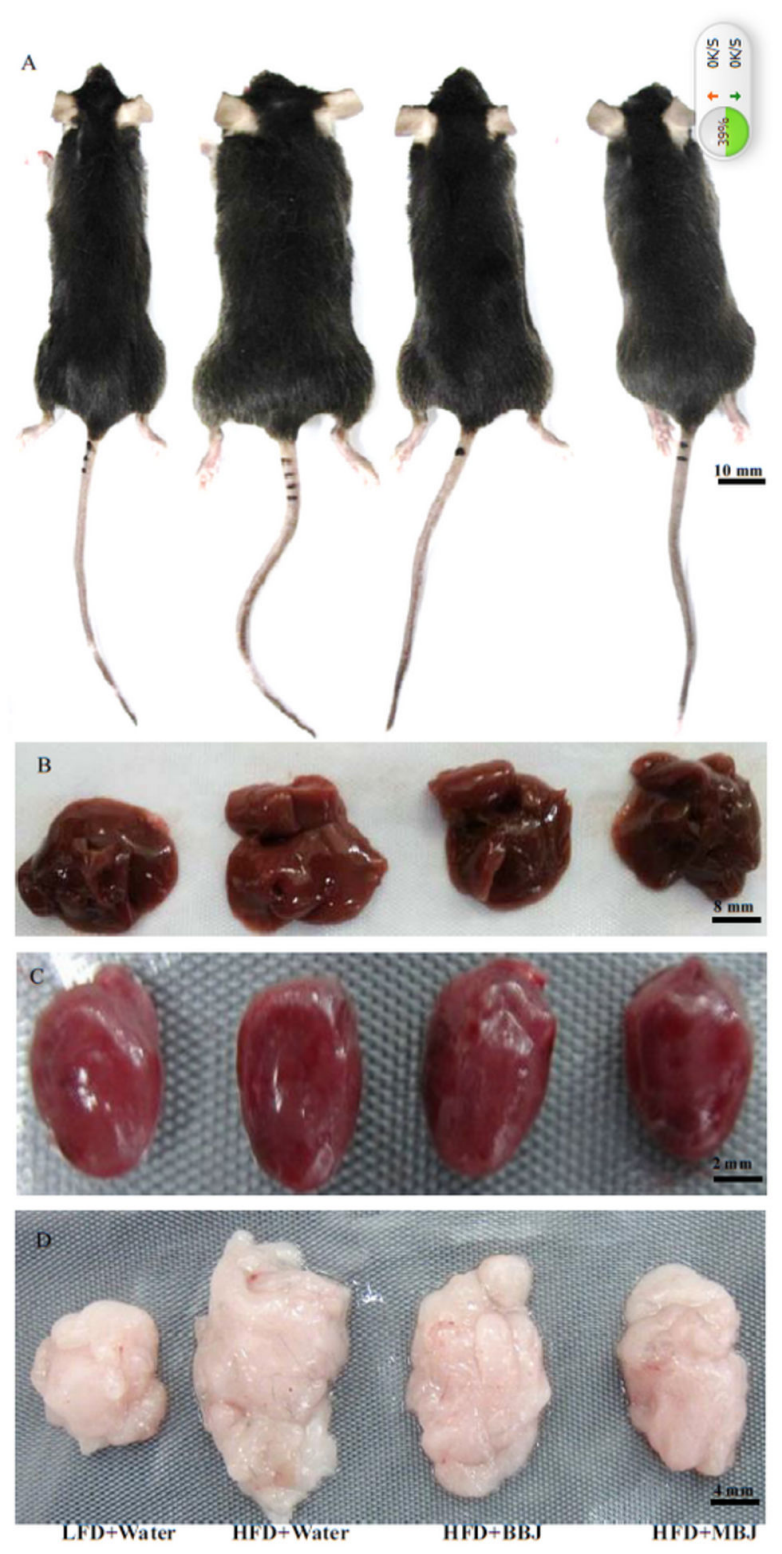
Representative macroscopic pictures of male C57BL/6 mice from different groups at the end of experiment. A, C57BL/6 mice; B, liver; C, heart; D, epididymal white adipose tissue.

### Histological analysis

 Liver and epididymal white adipose tissue samples were fixed with 10% formalin and then stained with oil red O and hematoxylin and eosin (H&E), respectively.

### Hepatic lipids

 The liver samples from each mouse were homogenized in PBS, and the total lipids were determined according to a previously described method [22]. The concentrations of liver triglycerides and total cholesterol were estimated using the same enzymatic kit for serum analysis.

### Quantitative real-time PCR

 Total RNA from liver and white adipose tissue were extracted with Trizol (Invitrogen Technologies, USA) according to the manufacturer’s protocol. The single-stranded cDNA was synthesized using the Transcriptor First Strand cDNA Synthesis Kit (Roche). Quantitative PCR was performed using LightCycler 480 system (Roche). The 20 μl reaction mixture was prepared as follows: 10 μl SYBR Green Quantitative PCR SuperMix-UDG (Invitrogen Technologies, USA), 0.4 μl of forward primer (10 μM), 0.4 μl of reverse primer (10 μM), and 2 μl of cDNA. The real-time PCR conditions were as follows: 95 for 10 min followed by forty five cycles at 95 °C for 15 s, 60 °C for 5 s; 72 °C for 15 s. The sequences primer used in the experiments were shown in Table 1. All the results were obtained from at least three independent experiments. The liver expression of PPARγ, FAS, ACO, CPT 1 and white adipose tissue expression of IL-1β, IL-6 and TNFα were examined and normalised using β-actin as an internal control.

**Figure 2:**
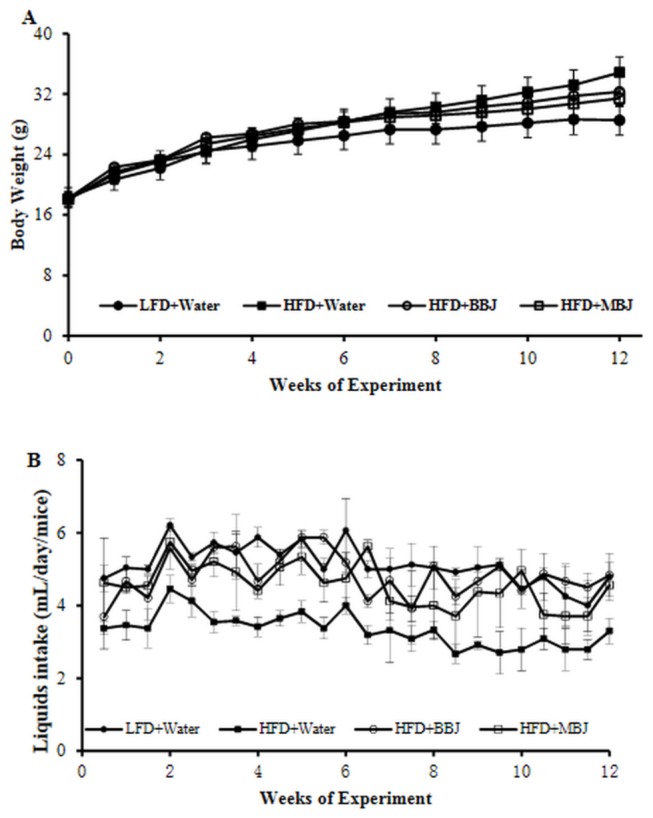
Body weight of male C57BL/6 mice consuming the indicated diets for the 12-week intervention period; B Liquids intake of mice during the intervention period. Values are mean ±SEM.

**Table 1:** Sequence of primers used in quantitative real-time PCR.

Gene	Sense Primer	Antisense (5’➡3’)
PPARγ	CGCTGATGCACTGCCTATGA	AGAGGTCCACAGAGCTGATTC
FAS	CTGAGATCCCAGCACTTCTTGA	GCCTCCGAAGCCAAATGAG
CPT 1	CGCACGGAAGGAAAATGG	TGTGCCCAATATTCCTGG
ACO	CTTGTTCGCGCAAGTGAGG	CAGGATCCGACTGTTTACC
IL-1β	GCTACCTGTGTCTTTCCCGT	CGTCACACACCAGCAGGTTA
IL-6	TCCAGTTGCCTTCTTGGGAC	GGTCTGTTGGGAGTGGTATCC
TNFα	AGCCCACGTCGTAGCAAACCAC	ACACCCATTCCCTTCACAGAGC
β-actin	ATGTGGATCAGCAAGCAGGA	AAGGGTGTAAAACGCAGCTCA

PPARγ, peroxisome proliferator-activated receptor; FAS, fatty acid synthase; CPT 1, carnitine palmitoyl transferase; ACO, acyl-CoA oxidase; IL-1β, iterleukin-1β; IL-6, iterleukin-6; TNFα, tumor necrosis factor α

### Statistical analysis

 The sample groups were statistically analyzed using SPSS 19.0 statistical software. Mean ± standard error for each group was calculated. Significant differences among groups were examined by post-hoc Duncan’s multiple range tests. P < 0.05 was considered significant.

## Results

### Anthocyanins components

The total polyphenol content of blueberry juice (BBJ) and mulberry juice (MBJ), as measured by the Folin-Ciocalteu assay, were 6.39 ± 3.23 mg GAE/ mL and 33.14 ± 1.24 mg GAE/ mL. Anthocyanins, as the major polyphenol, were also characterized. We identified nine kinds of anthocyanins in BBJ and four kinds of anthocyanins in MBJ (Table 2). In addition, the major anthocyanins in BBJ were petunidin-3-arabinoside, cyanidin-3-galactoside and delphinidin-3-galactoside, which was in agreement with the previous studies [14,17]; the predominant anthocyanins in MBJ were cyanidin-3-glucoside and cyanidin-3-rutinoside, which was consistent with previous studies [16,23]. 

**Table 2:** Mass spectrometry data and the content of identified anthocyanin in BBJ and MBJ.

Identified anthocyanins	m/z MS/MS	BBJ (mg/mL)	MBJ (mg/mL)
Cyanidin-3-glucoside	449/287		11.78
Cyanidin-3-galactoside	449/287	0.51	
Cyanidin-3-arabinoside	419/287	0.26	
Cyanidin-3-rutinoside	595/448/287		9.61
Delphinidin-3-glucoside	465/303	0.37	
Delphinidin-3-galactoside	465/303	0.67	
Delphinidin-3-arabinoside	435/303	0.44	
Petunidin-3-glucoside	479/317	0.17	
Petunidin-3-arabinoside	449/317	1.00	
Pelargonidin-3-glucoside	432/271		0.30
Pelargonidin-3-rutinoside	578/271/ 432		0.16
Malvidin-3-galactoside	493/331	0.19	
Malvidin-3-glucoside	463/331	0.47	
Total anthocyanins		4.09	21.86

Values are expressed as mean ± SEM

### Body weight, food intake and liquid consumption

 All the mice treated with BBJ and MBJ as the sole drinking vehicle for the whole 12 weeks were healthy. Initial body weight of mice averaged 18.10 g, which was not significant difference among four groups. After 12 weeks, the mice fed with HFD increased greater body weight compare with the control LFD mice (Figure 1A, Figure 2A). Intake of BBJ or MBJ reduced body weight for the HFD-fed mice by 7.3 % and 9.81 %，but their bodyweight was still higher than the mice in LFD group (Figure 2A). Furthermore, there were no statistically significant differences among four groups as to body length (Figure 1A, Table 3). 

**Figure 3:**
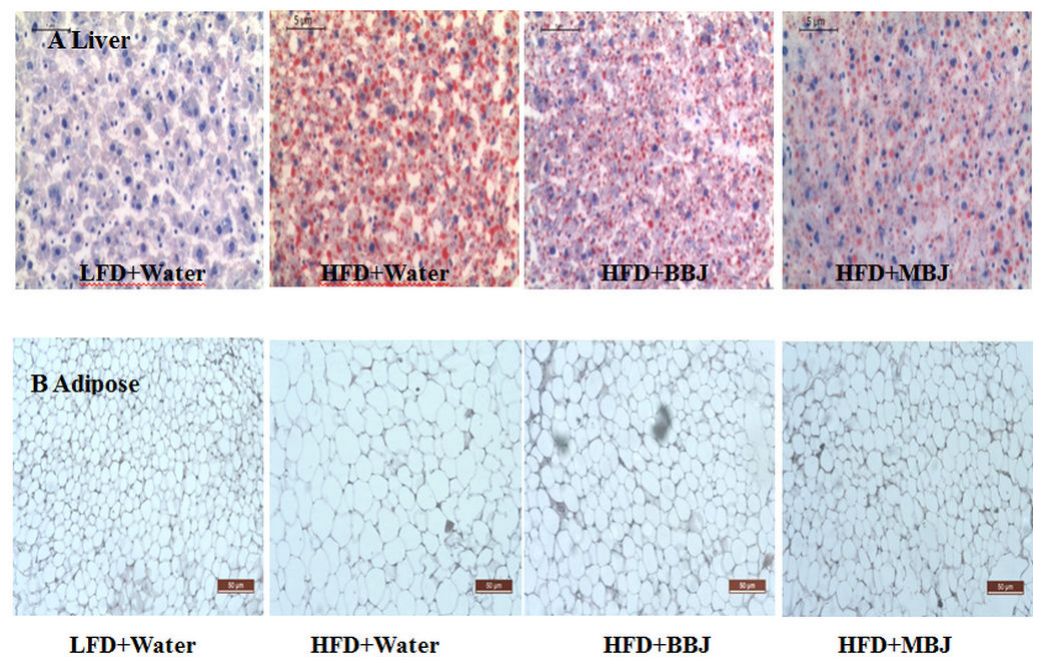
Morpholoogy changes in liver and epididymal adipose tissue for the male C57BL/6 mice. A, Oil Red O was used to stain livers sections of mice; B, H&E stained epididymal adipose tissue. Representative sections are from three mice from dietary group of mice.

**Figure 4:**
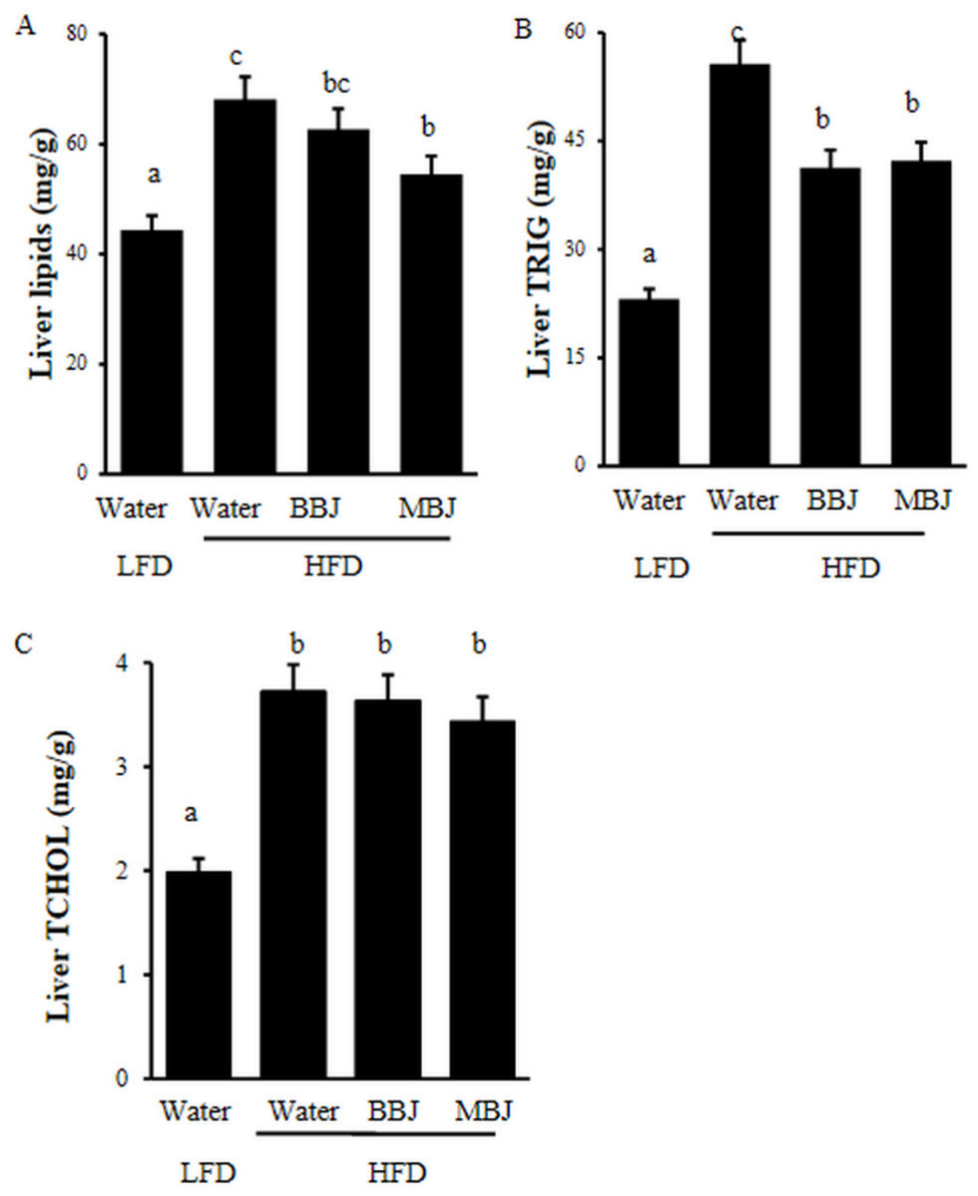
Hepatic contents of total lipid, triacylglycerol and cholesterol. Values are mean ±SEM. The means marked with superscript letters are significantly different relative to others.

**Table 3:** Tissue weight and serum parameters and Hepatic lipids for the male C57BL/6 mice in LFD+ Water, HFD+ Water, HFD+BBJ and HFD+MBJ group at the end of experiment.

item	LFD+Water	HFD+Water	HFD+BBJ	HFD+MBJ
Body length (snout-anus)	9.2±0.3	9.6±0.4	9.5±0.3	9.5±0.4
Tissue index (×100)				
Heart	0.51±0.05^b^	0.38±0.04^a^	0.48±0.02^b^	0.45±0.09^b^
Liver	4.02±0.08^c^	2.88±0.05^a^	3.32±0.32^b^	3.37±0.26^b^
Kidney	1.25±0.04^b^	0.97±0.11^a^	1.23±0.11^b^	1.21±0.09^b^
Epididymal WAT	1.93±0.07^c^	5.98±0.26^a^	5.87±0.55^a^	5.93±0.27^a^
Interscapular BAT	0.41±0.05 ^c^	0.34±0.03 ^a^	0.37±0.04 ^b^	0.38±0.07 ^b^
Serum				
ALT (U/L)	28.75±1.43^b^	35.25±1.03^a^	33.00±3.13^a^	23.42±2.21^c^
AST (U/L)	91.55±4.32^c^	132.75±5.32^a^	121.75±6.48^b^	96.78±7.32^c^
GLU (mmol/L)	5.94±0.51^b^	7.19±0.43^a^	6.44±0.56^a^	5.00±0.37^c^
TG (mmol/mL)	0.82±0.03^c^	1.67±0.11^a^	1.57±0.17^a^	1.07±0.03^b^
TCH (mmol/mL)	2.34±0.12^c^	4.42±0.23^a^	3.36±0.19^b^	3.25±0.20^b^
HDL-CH (mmol/mL)	2.18±0.11^c^	3.83±0.17^a^	2.97±0.16^b^	2.93±0.01^b^
LDL-CH (mmol/mL)	0.05±0.01 ^b^	0.17±0.03 ^a^	0.04±0.02^b^	0.06±0.02^b^

Values are expressed as mean ± SEM

ALT，aspartate aminotransferase；AST, alanine aminotransferase; GLU, serum glucose; TG, triglycerides; TCH, total cholesterol; HDL-CH, high-density lipoprotein cholesterol; LDL-C, low density lipoprotein cholesterol; WAT, white adipose tissue; BAT, brown adipose tissue.

The means marked with superscript letters are significantly different relative to others.

Liquid consumption by mice on the LFD group (5.13 ± 0.34 mL day^-1^ per mouse) was highest and on the HFD group (3.34 ± 0.46 mL day^-1^ per mouse) was lowest (Figure 2B). The average consumption of the BBJ and MBJ were 4.83 ± 0.36 and 4.56 ± 0.57 mL day^-1^ per mouse. As fruit juice has higher energy content compared with water [11], the consumption of BBJ and MBJ corresponds to an excess of energy intake of 1.3 and 1.8 kcal day^-1^. In spite of this, none of juices provide altered food consumption, as indicated by the daily food intake that was ^~^3.2 g per day throughout the experiment. 

Weights of heart, liver, kidney and interscapular brown adipose tissue did not change due to dietary treatment (Figure 1, Table 3). However, when expressed as a percentage of body weight, heart, liver, kidney and interscapular brown adipose tissue were smaller in HFD-fed mice compared to LFD-fed mice (Table 3). The weight of epididymal fat was much higher in the HFD-fed mice compared to control LFD-fed mice, but its weight would be decreased after administration with BBJ or MBJ.

### Serum parameters

 Mice in HFD group showed elevation in serum glucose, triglyceride and cholesterol levels compared to LFD group (Table 3). BBJ decreased the levels of serum glucose and cholesterol level slightly, while MBJ significantly affected serum glucose. 

**Figure 5:**
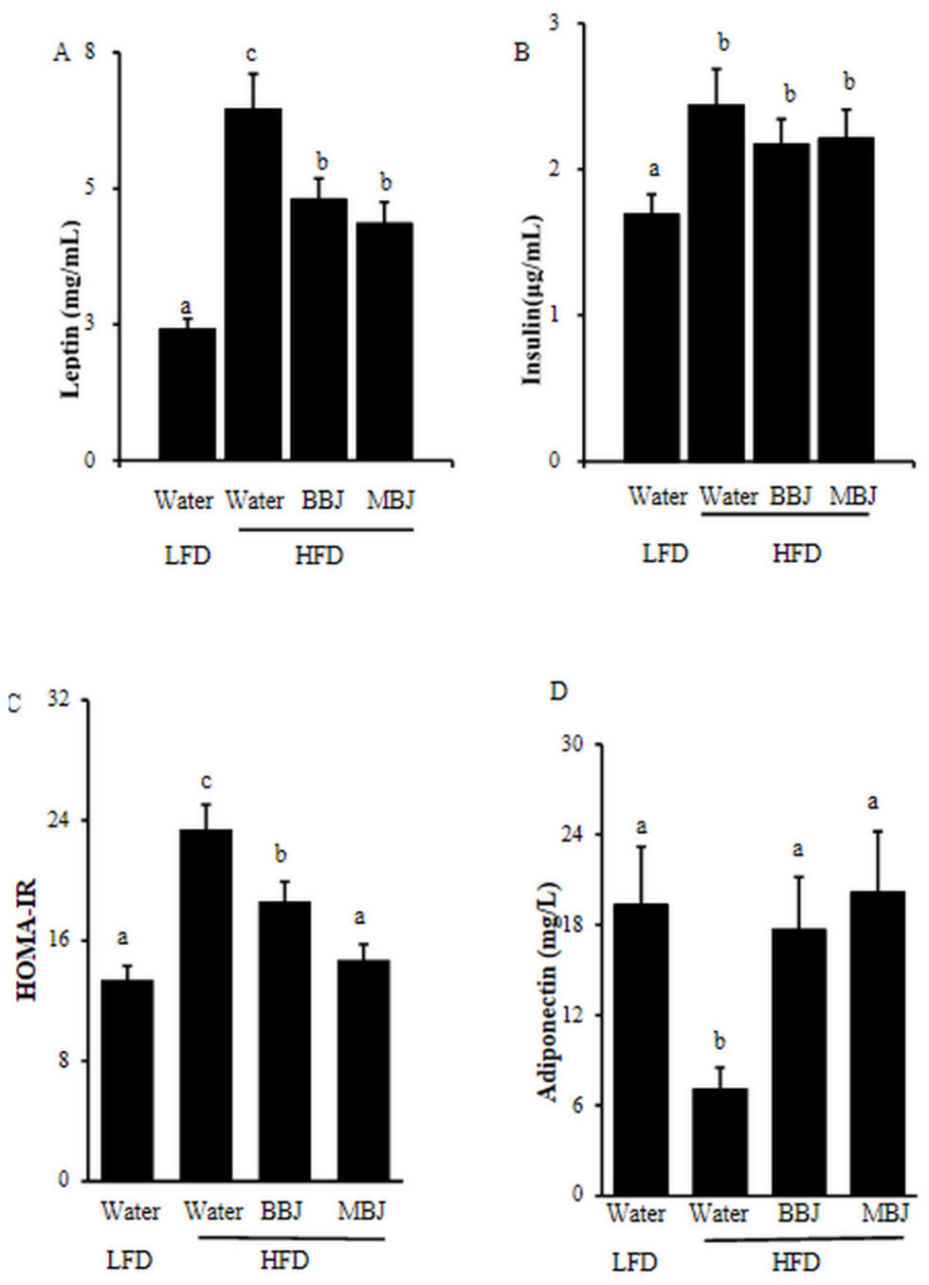
Serum insulin levels, leptin levels, HOMA-IR and adiponectin in mice. Values are mean ±SEM. The means marked with superscript letters are significantly different relative to. others.

### Liver and adipose tissue morphology

 Mice fed with HFD showed intense lipid accumulation in the liver (Figure 3A). In contrast, BBJ or MBJ significantly alleviated the lipid accumulation in those HFD-fed mice. Figure 3B displays the histology of epididymal white adipose tissue of mice. The mice fed with HFD showed hypertrophy of the adipocytes in the adipose tissue. The phenotype of adipocytes was attenuated when those HFD-fed mice were treated with BBJ or MBJ (Figure 1D, Figure 3B).

**Figure 6:**
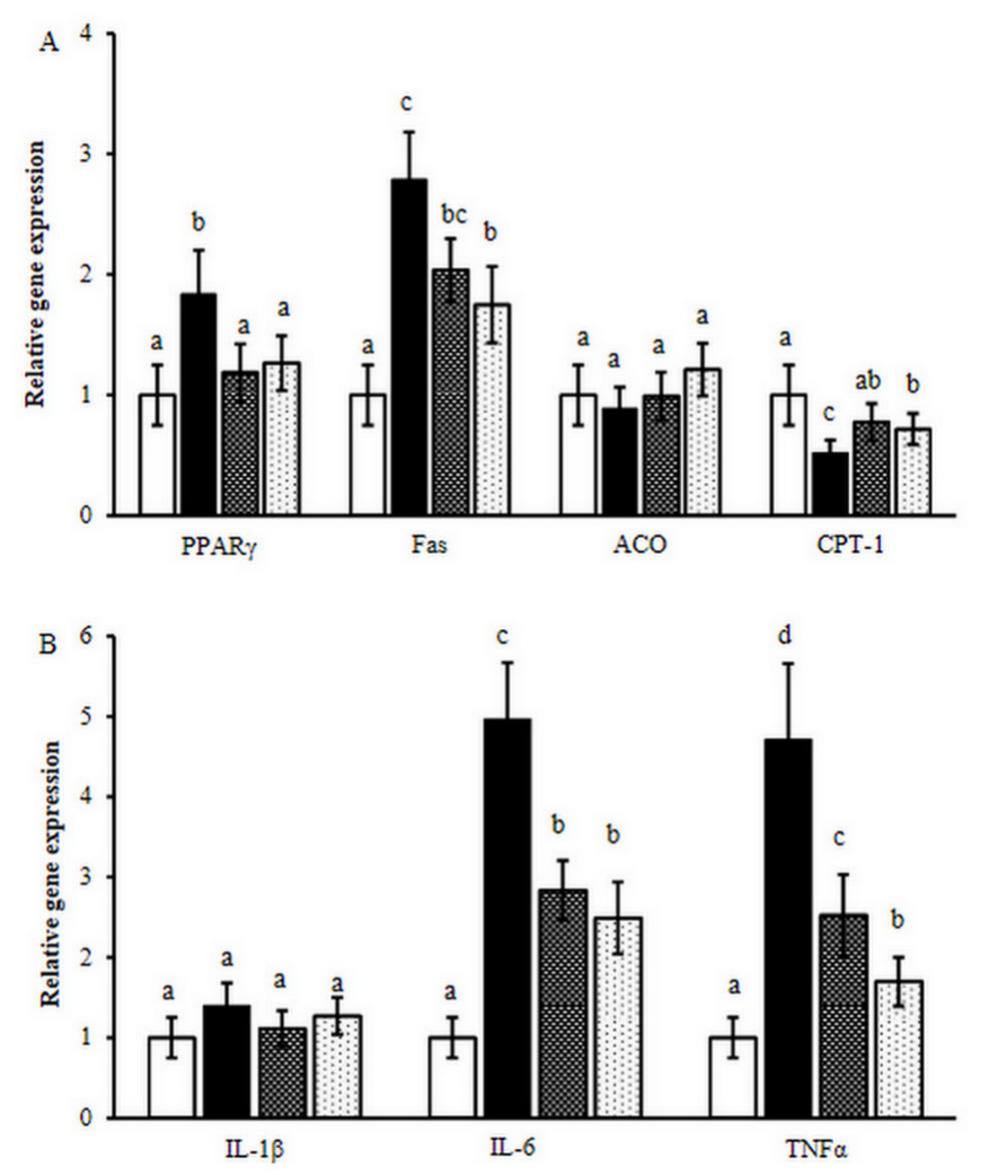
Gene expression determained by quantitative real-time PCR. A, Expression of PPARγ, FAS, CPT 1, ACO in the liver tissue; B Expression of IL-1β, IL-6, TNFα in the epididymal adipose tissue. LFD (□), HFD (■), HFD with BBJ (▓), HFD with MBJ (░). The means marked with superscript letters are significantly different relative to others.

### Hepatic lipids

Figure 4 exhibits the hepatic content of total lipid, triacylglycerol and cholesterol elevate for all mice fed with HFD control relative to the LFD control. BBJ did not alter the contents of liver lipids and cholesterol, while significantly decreased the levels of liver triacylglycerol. MBJ significantly attenuated the HFD induced liver lipids. 

### Leptin, insulin and adiponectin

Serum leptin and insulin levels in mice serum were examined (Figure 5). Serum leptin and insulin level were elevated in mice fed HFD. BBJ or MBJ significantly lowered serum leptin levels compared to the HFD control (Figure 5). Although BBJ and MBJ were unable to alter the insulin secretion, the HOMA-IR was significantly reduced.

The concentration of adiponectin was higher in the LFD group when compared to the groups that received high-fat diet. Both of juices elevated the adiponectin levels respectively. 

### Molecular biological observation of liver and white adipose tissue

The mRNA expression levels of PPARγ, FAS, ACO and CPT 1 were determined in liver tissue (Figure 6A). Quantitative real-time PCR analysis was also performed to evaluate the expression of IL-1β, IL-6 and TNFα (Figure 6B). As compared to the LFD group, mice fed with HFD caused an up-regulation of PPARγ, FAS, IL-6 and TNFα genes, and a down-regulation of CPT 1 gene. BBJ or MBJ markedly reduced the expression levels of PPARγ, FAS, IL-6 and TNFα compared to the HFD control, while increased the CPT 1 expression levels. 

## Discussion

 Many epidemiological studies have shown the benefits of a diet rich in fruit and vegetables to human health and for the prevention of various degenerative diseases, such as cancer and cardiovascular diseases [24]. Anthocyanins are natural components of the human diet, as they are present in many vegetables and fruits, especially in berries. They have attracted research interest because of their health functions such as anti-obesity properties [8,24]. Blueberry and mulberry are known to be particularly rich in anthocyanins, which have been found to have beneficial effects for people with obesity and metabolic syndrome [14,17]. However, the juices consumption on weight gain is still controversial mainly owing to its sugar content. In this study, we explored the effects of consumption of blueberry and mulberry juice on obesity development in mice fed a low fat diet (LFD) or a high fat diet (HFD). 

As expected, the present investigation confirmed that HFD could induce a great gain of body weight, an increase of serum and liver lipids, as well as an elevation of insulin and leptin levels [25,26]. Our results are in agreement with previous experimental data suggesting that the effect of anthocyanins administration both in diet-induced and genetic models of obesity is the reduction of body weight [10,16]. In this respect, Salamone et al. showed that *Moro* orange juice (rich in anthocyanins) limited body weight gain and exerted beneficial effects on several metablic aspects related to obesity in mice fed HFD [27]. Similarly, Titta et al. found blood orange juice inhibited fat accumulation in C57/BL6 mice [11]. 

C57BL/6 mice are susceptible to diet-induced obesity [25]. In this study, the mice showed high lipid accumulation in the liver (Figure 3A). HFD significantly increased triglycerides in the liver (Figure 4), but BBJ and MBJ countered this effect. Although the effects of body fat level of mice on the response to BBJ and MBJ remain unknown, BBJ and MBJ consumption may regulate lipid metabolism by suppressing the fatty acid synthesis related gene (PPARγ and FAS) and inducing the expression of β-oxidation–related gene (CPT 1). 

Animal studies have shown that C57BL/6 mice fed with an HFD supplemented with anthocyanins exhibit improved insulin resistance [10,14,16]. In the present study, insulin resistance (assessed by HOMA-IR) was slightly observed in the HFD-fed mice, BBJ and MBJ intake reduced resistance to insulin resistance (Figure 5). 

Leptin is a product of an obese gene secreted by adipose tissues and has an important function in lipid metabolism [28]. Studies have suggested that obese models exhibit high serum leptin concentrations [29], which decrease when treated with anthocyanins. A similar observation was found in our study (Figure 5). In the present study, HFD could increase the weight of the epididymal fat (Figure 1D, Table 3) and induce hypertrophy in adipocytes (Figure 3B). BBJ and MBJ supplementation could decrease the epididymal fat and the size of adipocytes. BBJ and MBJ may directly affect both the number and the size of adipocytes in adipose tissues, and this observation is likely associated with leptin production.

Adiponectin is an adipocyte secretory protein hormone that modulates metabolic processes such as fatty acid oxidation and glucose regulation [30,31]. The circulating levels of adiponectin decreased in obese subjects. Increased concentration of adiponectin hormone was related with a reduction of bodyweight in obese animals [32,33]. In tandem with the increased concentration of adiponectin, our results also showed that BBJ and MBJ potentially induced fatty acid oxidation and reduced serum glucose levels (Figure 5D). 

Obesity is associated with a state of chronic or low grade systemic inflammation which increases production of obesity-related inflammatory cytokines, such as IL-1β, IL-6, TNFα, leptin and decrease anti-inflammatory cytokine levels, such as adiponectin [1,34-36]. In this study, we found HFD fed mice was under the pathophysiologic condition of inflammation associated with obesity evidenced by high levels of IT-6, TNFα and leptin. Our results further indicated that BBJ and MBJ exerted potentially anti-inflammatory effect (Figure 5A and Figure 6B)

In summary, BBJ and MBJ suppressed the body weight gain of the HFD-fed C57BL/6 mice. Intake of BBJ or MBJ reduced body weight for the HFD-fed mice by 7.3% and 9.81 %. Furthermore, BBJ or MBJ supplementation could significantly decrease TG in the liver, and inhibit leptin secretion. BBJ or MBJ supplementation could also improve insulin resistance. Moreover, BBJ and MBJ exerted potentially anti-inflammatory effect. Therefore, BBJ and MBJ could be used to help counteract obesity. 
